# Single-cell analysis of mouse uterus at the invasion phase of embryo implantation

**DOI:** 10.1186/s13578-022-00749-y

**Published:** 2022-02-05

**Authors:** Jia-Peng He, Qing Tian, Qiu-Yang Zhu, Ji-Long Liu

**Affiliations:** Guangdong Laboratory for Lingnan Modern Agriculture, College of Veterinary Medicine, South China Agricultural University, Tianhe District, No. 483 Wushan Road, Guangzhou, 510642 China

**Keywords:** Embryo implantation, Mouse, Single-cell RNA-seq, Transcriptional changes

## Abstract

**Background:**

Embryo implantation into the uterus is a crucial step for human reproduction. A hypothesis has been proposed that the molecular circuit invented by trophoblasts for invasive embryo implantation during evolution might be misused by cancer cells to promote malignancy. Unfortunately, our current understanding of the molecular mechanism underlying embryo implantation is far from complete.

**Results:**

Here we used the mouse as an animal model and generated a single-cell transcriptomic atlas of the embryo implantation site of mouse uterus at the invasion phase of embryo implantation on gestational day 6. We revealed 23 distinct cell clusters, including 5 stromal cell clusters, 2 epithelial cell clusters, 1 smooth muscle cell cluster, 2 pericyte clusters, 4 endothelial cell clusters, and 9 immune cell clusters. Through data analysis, we identified differentially expression changes in all uterine cell types upon embryo implantation. By integrated with single-cell RNA-seq data from E5.5 embryos, we predicted cell–cell crosstalk between trophoblasts and uterine cell types.

**Conclusions:**

Our study provides a valuable resource for understanding of the molecular mechanism of embryo implantation.

**Supplementary Information:**

The online version contains supplementary material available at 10.1186/s13578-022-00749-y.

## Background

Embryo implantation in humans is interstitial. It consists of the following three phases: embryo apposition, attachment, and invasion. Immediately after apposition and attachment to endometrial epithelium, the blastocyst penetrates through the epithelium, followed by the basal lamina, and invades into the stroma [1]. It has been well established that excessive trophoblast invasion may lead to the pathogenesis of placenta creta [2], while shallow trophoblast invasion may result in pre-eclampsia and intrauterine growth restriction [3]. Interestingly, it has long been recognized that there are striking similarities between trophoblast cells and invasive cancer cells, both of which share proliferative, migratory and invasive properties, as well as the capacity to confer immune privilege [4]. A hypothesis has been proposed that the molecular circuit invented by trophoblasts for invasive embryo implantation during evolution might be misused by cancer cells to promote malignancy [5]. Unfortunately, our current understanding of the mechanisms underlying embryo implantation is far from complete.

Due to ethical restrictions and experimental difficulties, in vivo analysis of embryo implantation heavily relies on mice [6]. Slightly different from humans, embryo implantation in mice is eccentric. The apposition phase of mouse embryo implantation is thought to occur from the morning to the midnight of gestational day 4 (GD4). During this phase, the embryo seeks its position on the luminal epithelium of uterus. After that, the embryo attaches to the receptive uterine epithelium. A firm connection between the blastocyst and uterine luminal epithelium is established on the morning of GD5. The invasion phase of embryo implantation occurs on GD6, when the blastocyst penetrates through the epithelium and invades into the stroma [7, 8]. Previously, two studies have analyzed global gene expression changes associated with the invasion phase of embryo implantation in mice by using high-throughput transcriptomic approaches [9, 10]. The limitation of both studies was that the whole uterus was used. The uterus is a complex structure consisting of many cell types, including luminal and glandular epithelial cells, stromal cells, smooth muscle cells, endothelial cells, and various immune cells [11]. Thus, whole uterus transcriptomic studies were unable to accurately capture cell-type-specific gene expression changes.

In the present study, by using the-state-of-the-art single-cell RNA-seq approach [12], we resolved all cell types at the embryo implantation site of the mouse uterus on GD6. Through data analysis, we identified differentially expression changes in all cell types at the invasion phase of embryo implantation. We also predicted cell–cell crosstalk between trophoblasts and uterine cell types. Our study provides a valuable resource for understanding of the molecular mechanism of embryo implantation.

## Materials and methods

### Sample collection

Adult CD-1 mice of the SPF grade were used in this study. All mice were caged under light-controlled conditions (14 h/10 h light/dark cycles) with free access to regular food and water. Female mice were mated with fertile males and success of mating was confirmed the next morning by the presence of a vaginal plug. The day of the vaginal plug was denoted as gestation day 1 (GD1). On GD6, the implantation sites and inter-implantation sites (served as a control) were collected separately. All animal procedures were approved by the Institutional Animal Care and Use Committee of South China Agricultural University (No. 2020B078, approved on 29/09/2020).

### Bulk RNA-seq analysis

The total RNAs from uterine tissues were extracted with the TRIzol reagent (Invitrogen). RNA-seq libraries were generated by using the TruSeq RNA sample preparation kit (Illumina) and sequenced on an Illumina HiSeq 2500 system. Using UCSC mm10 mouse genome as reference, raw data were analyzed using TopHat v2.0.4 [13] and Cufflinks v2.2.1 [14] as described previously [15]. Gene expression levels were measured as fragments per kilobase per million (FPKM).

### Single-cell dissociation of mouse uterus

Single-cell suspension was prepared as described previously [16, 17]. Briefly, the uterine tissues from 3 mice for each group were pooled and minced with a blade. Tissues were then incubated in dissociation buffer containing 2 mg/ml Collagenase II (#C6885, Sigma-Aldrich), 10 mg/ml Dispase II (#354,235, Corning) and 50,000 U/ml DNase I (#DN25, Sigma-Aldrich) for up to 30 min at 37 °C in a shaking incubator. The digestion progress was checked every 5 min with a microscope until a single cell suspension was achieved. The single-cell suspension was then passed through a 40-μm cell strainer to remove undigested tissues. Cells were spun down at 250 g at 4 °C for 4 min and the pelleted cells were washed using centrifugation. In order to measure cell viability, cells were strained with AO/PI solution (#CS2-0106, Nexcelom Bioscience) and counted using a Cellometer Auto 2000 instrument (#SD-100, Nexcelom Bioscience). The single-cell suspension was carried forward to single-cell RNA-seq only if the cell viability was > 80% and the percentage of cell clumps was < 10%.

### Single-cell RNA-seq library preparation and sequencing

The final concentration of single-cell suspension was adjusted to 1000 cells/μl and a volume of 15 µl was loaded into one channel of the ChromiumTM Single Cell B Chip (#1,000,073, 10 × Genomics), aiming at recovering 8000–10,000 cells. The Chromium Single Cell 3' Library & Gel Bead Kit v3 (#1,000,075, 10 × Genomics) was used for single-cell bar-coding, cDNA synthesis and library preparation, following the manufacturer's instructions provided as the Single Cell 3' Reagent Kits User Guide Version 3. Library sequencing was performed on an Illumina novaseq 6000 system configured with the paired-end 150-bp protocol at a sequencing depth of approximately 400 million reads.

### Single cell RNA-seq data analysis

Raw data bcl files from the Illumina NovaSeq 6000 platform were converted to fastq files using the bcl2fastq tool (v2.19.0.316). These fastq files were aligned to the mm10 mouse reference genome by using the CellRanger software (v3.0.1, 10 × Genomics). The resulting gene counts matrix was analyzed with the R package Seurat (v3.1.3) [18]. Cell with fewer than 200 or greater than 6000 unique genes, as well as cells with greater than 25% of mitochondrial counts, were excluded. Meanwhile, genes expressed in fewer than 3 cells were removed. Following data filtering, the gene counts matrix was normalized and scaled by using NormalizeData and ScaleData, respectively. The top 2000 highest variable genes were used for the principal component analysis (PCA) and the optimal number of PCA components was determined by the JackStraw procedure. Single cells were clustered by the K-nearest neighbor (KNN) graph algorithm in PCA space and visualized using the t-distributed stochastic neighbor embedding (tSNE) dimensional reduction technique. The cell type label for each cell cluster was manually assigned based on canonical cell markers. The FindAllMarkers function was used to identify novel marker genes for each cluster with a minimum of 10% of cells expressing the gene within the cluster and a minimum logFC threshold of 0.25. In order to find differential expressed genes in the same cell type between pre-receptive uterus and receptive uterus, the FindMarkers function in Seurat was used with min.logfc being set to 0.25 and min.pct being set to 0.20.

### Pseudotime analysis

Monocle2 package v2.18.0 [19] was used for pseudotime analysis. The count data and meta data were export from the Seurat object and then import into the CellDataSet object in Monocle2. Feature genes were selected by using the differentialGeneTest function. After The dimension reduction by using the DDRTree algorithm, the orderCells function was used to infer the trajectory with default parameters. The reconstructed trajectory was visualized by the plot_cell_trajectory function.

### Gene ontology analysis

Gene ontology (GO) analysis was performed as described previously [20]. GO terms were grouped according to the biological process category in the Mouse Genome Informatics (MGI) GOslim database [21]. To test for enrichment, a hypergeometric test was conducted and P < 0.05 was used as significance threshold to identify enriched GO terms.

### Pathway analysis

Pathway enrichment analysis was conducted by using the Metascape v7.4 online tools [22]. The significance threshold for FDR was set at 0.05.

### Cell–cell communication analysis

The CellChat v1.1.0 software [23] was used to infer cell–cell communication based on ligand-receptor interaction with default parameters. For each ligand-receptor pair, CellChat assigned a communication probability value by the law of mass action based on the average expression values of a ligand by one cell group and that of a receptor by another cell group. The statistical significance of communication probability values was assessed by a permutation test. P < 0.05 were considered statistically significant.

## Results

### A single-cell atlas of mouse uterus on gestational day 6

**Fig. 1:**
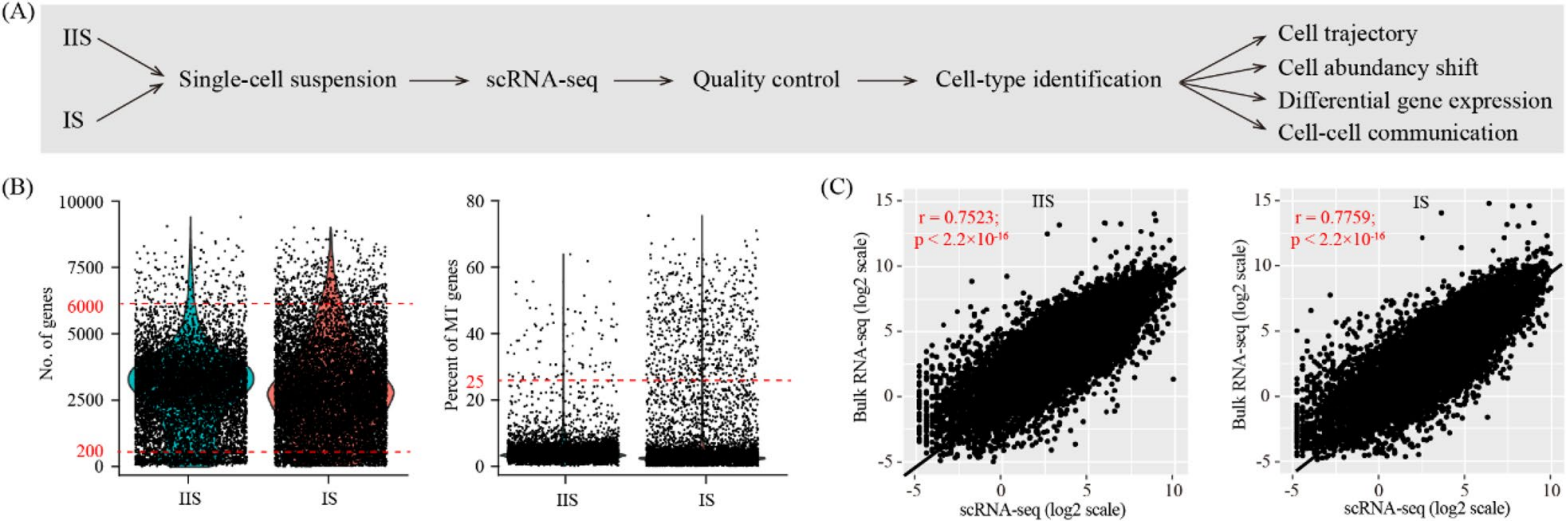
Single-cell transcriptome analysis of mouse uterus on gestational day 6. **A** A flowchart overview of this study. IIS, inter-implantation site; IS, implantation site. **B** Single-cell RNA-seq data quality control. Cells with detected genes fewer than 200 or more than 6000 were removed (left) and only cells with total mitochondrial gene expression below 25% were kept (right). **C** Scatter plots showing the correlation between single-cell RNA-seq and bulk RNA-seq. For single-cell RNA-seq data, gene expression levels were averaged and normalized as transcript per million (TPM). For bulk RNA-seq data, gene expression levels were measured as fragments per kilobase per million (FPKM)

To create a cell-type resolved map of mouse uterus at the invasion phase of embryo implantation, we performed single-cell RNA-seq analysis (Fig. 1A). The implantation site (IS) and the inter-implantation site (IIS) of mouse uterus were collected on gestational day 6 (GD6). The whole uterus, which is consist of endometrium, myometrium and perimetrium, was used. The embryo at IS was also kept. Single-cell RNA-seq data were generated by using the 10× Genomics platform. After quality control, a total of 16,257 cells (7065 for IIS and 9192 for IS) were obtained (Fig. 1B). In order to validate this single-cell RNA-seq dataset, we also generate a bulk RNA-seq dataset using the same samples. It turned out that the cell-averaged single-cell RNA-seq data were highly accordant with the conventional bulk RNA-seq data (r = 0.7523 for IIS and r = 0.7759 for IS), indicative of high quality of our single-cell RNA-seq data (Fig. 1C).

**Fig. 2:**
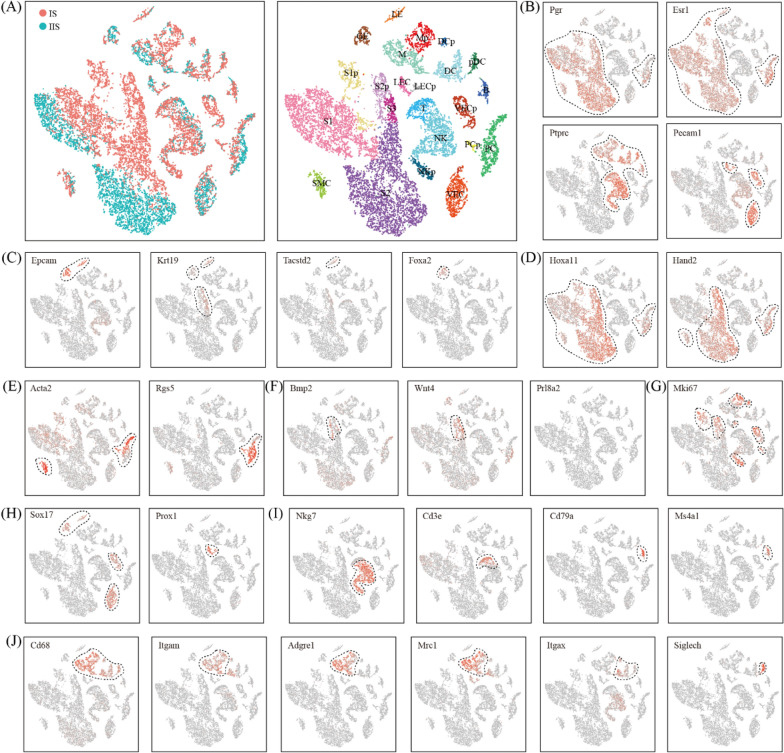
Identification of different cell types by using canonical marker genes. **A** The t-Stochastic neighbor embedding (tSNE) representation of single-cell RNA-seq data obtained from IS and IIS of the uterus. Single cells were grouped by cellular origin (right) and cell clusters (left). LE, luminal epithelial cells; GE, glandular epithelial cells; S1, deep stromal cells; S1p, proliferating deep stromal cells; S2, superficial stromal cells; S2, proliferating superficial stromal cells; S3, primary decidualization zone cells; SMC, smooth muscle cells; PC, pericytes; PCp, proliferating pericytes; VEC, vascular endothelial cells; VECp, proliferating vascular endothelial cells; LEC, lymphatic endothelial cells; LECp, proliferating lymphatic endothelial cells; NK, natural killer cells; NKp, proliferating natural killer cells; T, T cells; B, B cells; M, macrophages; Mp, proliferating macrophages; DC, dendritic cells; DCp, proliferating dendritic cells; pDC, plasmacytoid dendritic cells. (B-J) The expression pattern of canonical marker genes projected onto TSNE plots. Shown are pan-marker genes for hormone-responsive cells, immune cells and endothelial cells **B**, epithelial cells **C**, stromal cells **D**, smooth muscle cells and pericytes **E**, decidual cells **F**, proliferating cells **G**, endothelial cells **H**, lymphocytes **I**, and myeloid cells **J**. Dashed lines denote the boundaries of the cell cluster of interest

Unsupervised clustering analysis revealed 23 distinct cell clusters for all cells from IIS and IS combined (Fig. 2A). Major cell types were defined using the expression of known cell type-specific genes, with hormone-responsive cells expressing Pgr and Esr1 [24, 25], endothelial cells expressing Pecam1 [26] and immune cells expressing Ptprc [27] (Fig. 2B).

Hormone-responsive cells included epithelial cells expressing Epcam and Krt19 [28] (Fig. 2C), stromal cells expressing Hoxa11 [29] (Fig. 2D), smooth muscle cells expressing Acta2 [11] and pericytes expressing Rgs5 [30] (Fig. 2E). We found 2 epithelial cell clusters, LE and GE. LE was luminal epithelial cells expressing Tacstd2 and GE was glandular epithelial cells expressing Foxa2 [31]. We identified 5 stromal cell clusters, S1, S1p, S2, S2p and S3. Cells in S2 but not S1 expressed high levels of Hand2, implying that S2 was superficial stromal cells and S1 was deep stromal cells [32]. S3 was primary decidual zone stromal cells expressing decidualization marker genes Wnt4 [33] (Fig. 2F). S1p and S2p were a subset of proliferating S1 and S2 with high level of Mki67, respectively (Fig. 2G). Only 1 smooth muscle cell cluster, SMC, was found. Meanwhile, 2 pericyte clusters, PC and its proliferating subset PCp, were identified. Endothelial cells had 4 clusters: VEC and its proliferating subset VECp are vascular endothelial cells expressing Sox17, while LEC and its proliferating subset LECp are lymphatic endothelial cells expressing Prox1 [26] (Fig. 2H). There were 9 immune cell clusters (Fig. 2I, J). Included were natural killer cells (NK, Ptprc^+^Nkg7^+^Cd3e^−^) [27], proliferating natural killer cells (NKp, Ptprc^+^Nkg7^−^Cd3e^+^Mki67^+^), T cells (T, Ptprc^+^Nkg7^−^Cd3e^+^) [27], B cells (B, Ptprc^+^Cd79a^+^Ms4a1^+^) [27], macrophages (M, Ptprc^+^Adgre1^+^) [34], proliferating macrophages (M, Ptprc^+^Adgre1^+^Mki67^+^), dendritic cells (DC, Ptprc^+^Itgax^+^) [34], proliferating dendritic cells (DC, Ptprc^+^Itgax^+^Mki67^+^) and plasmacytoid dendritic cells (pDC, Ptprc^+^Siglech^+^) [35].

Finally, we aimed to discover novel markers for each cell type. We selected genes that were expressed significantly higher in the cell type of interest than the other cell types by Wilcoxon rank sum test. A complete list of these marker genes was presented in Additional file 1: Table S1.

### Reconstruction of developmental trajectory for primary decidual zone

**Fig. 3:**
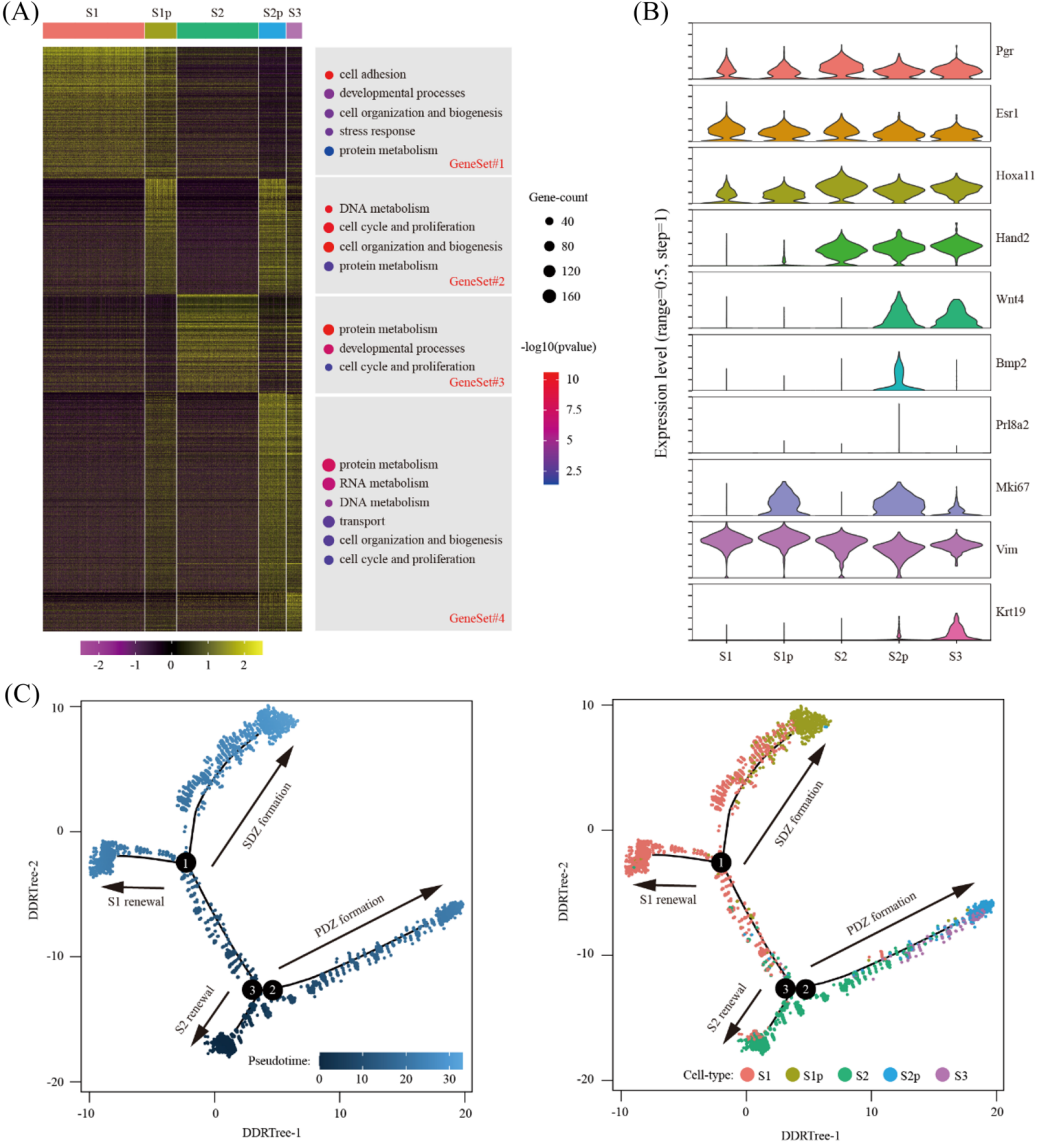
Developmental trajectory of primary decidual zone. **A** Heatmap of gene expression signatures for all stromal cell clusters (left). All signature genes were divided into 4 gene sets and enriched gene ontology terms were assigned accordingly (right). **B** The expression pattern of marker genes for clusters of stromal cell by violin plot. Shown were pan-stromal cell markers (Pgr, Esr1, Hoxa11 and Hand2), decidual cell markers (Wnt4 and Bmp2 and Prl8a2), proliferating cell marker (Mki67), mesenchymal marker (Vim) and epithelial marker (Krt19). **C** Pseudotime ordering of stromal cells. The distribution of pseudotime (left) and cell type (right) across the reconstructed trajectory were displayed. PDZ, primary decidual zone; SDZ, secondary decidual zone

In our single-cell RNA-seq data, we identified 5 clusters of stromal cells: S1 (deep stromal cells), S1p (proliferating deep stromal cells), S2 (superficial stromal cells), S2p (proliferating superficial stromal cells) and S3 (primary decidual zone stromal cells, PDZ). We selected signature genes for each cell cluster by using Wilcoxon rank sum test. After the removal of redundancy, we identified a total of 1784 signature genes (Additional file 2: Table S2). Through heatmap, we grouped all these signature genes into 4 gene sets (Fig. 3A). Gene set 1 with 403 genes were S1-specific. Gene ontology analysis showed that these genes were enriched in cell adhesion (P = 4.20 × 10^–11^), developmental processes (P = 1.10 × 10^–4^), cell organization and biogenesis (P = 3.27 × 10^–4^), stress response (P = 4.88 × 10^–4^) and protein metabolism (P = 3.87 × 10^–2^). Gene set 2 with 352 genes were decreased in S3 compared to its intermediate S2p. These genes were enriched in DNA metabolism (P = 1.00 × 10^–11^), cell cycle and proliferation (P = 1.00 × 10^–11^), cell organization and biogenesis (P = 2.59 × 10^–11^) and protein metabolism (P = 3.97 × 10^–3^). Gene set 3 of 300 genes were S2-specific. Based on GO, enriched terms were protein metabolism (P = 3.39 × 10^–11^), developmental processes (P = 6.05 × 10^–8^) and cell cycle & proliferation (P = 8.09 × 10^–3^). Gene set 4 of 729 genes were unchanged or increased in S3 compared to its intermediate S2p. Enriched GO terms were protein metabolism (P = 2.49 × 10^–8^), RNA metabolism (P = 3.23 × 10^–7^), DNA metabolism (P = 4.42 × 10^–5^), transport (P = 1.96 × 10^–3^) cell organization and biogenesis (P = 2.90 × 10^–5^), and cell cycle and proliferation (P = 4.91 × 10^–3^).

The expression of known marker genes for PDZ (S3) were examined. We found that PDZ expressed pan-stromal cell markers Pgr, Esr1 and Hoxa11, as well as the superficial stromal cell marker Hand2. Additionally, PDZ expressed decidualization marker Wnt4, but not Prl8a2 [36]. PDZ ceased proliferation from its intermediate S2p, showing low expression of Mki67. Interestingly, although PDZ expressed mesenchymal marker Vim, they were also positive for epithelial marker Krt19 (Fig. 3B).

To further reveal the relationship between these 5 stromal cell clusters, pseudotime trajectory analysis was conducted. Cells were arranged in a pseudotime manner with a pedigree reconstruction algorithm for biological processes based on transcriptional similarity. We found 2 paths of interest: (1) primary decidual zone formation, i.e. S2- > S2p/S3; and (2) secondary decidual zone formation, i.e. S1- > S1p (Fig. 3C).

### Cell–cell communication between primary decidual zone and trophoblast giant cells

**Fig. 4:**
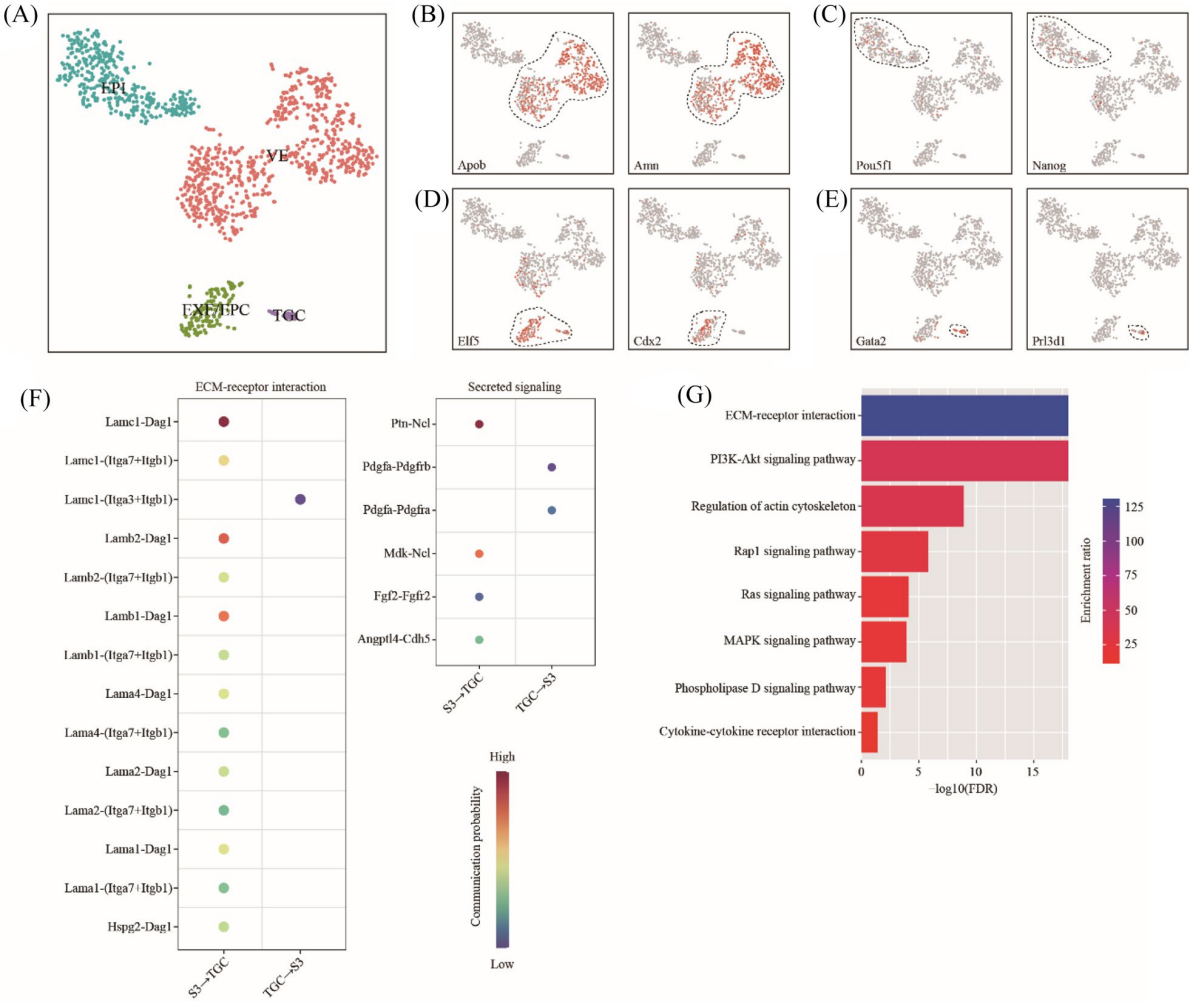
Cell–cell communication between primary decidual zone and trophoblast giant cells. **A** A single-cell atlas of E5.5 embryos which were collected from GD6 uterus from a previous study. EPI, epiblast; VE, visceral endoderm; EXE/EPC, extraembryonic ectoderm and ectoplacental cone; TGC, trophoblast giant cells. **B**–**E** TSNE map showing the expression pattern of well-known marker genes. **F** Dot plot showing selected ligand-receptor interactions underlying the crosstalk between primary decidual zone (S3) and trophoblast giant cells (TGC). The communication probability defined by the CellChat software were indicated by color. **G** KEGG Pathway enrichment analysis of ligand-receptor pairs by using the Metascape online tools

The cell–cell communication between primary decidual zone and trophoblast giant cells represents the key mechanism of embryo implantation. However, due to the relatively small number of embryonic cells at the implantation site, we did not find any embryo-related cell clusters in our single-cell RNA-seq data. Alternatively, we re-analyzed a published single-cell RNA-seq dataset on mouse E5.5 blastocysts [37]. E5.5 is equivalent to gestational day 6 in our study. By using canonical marker genes, 4 major cell types were identified (Fig. 4A). The 4 cell clusters were visceral endoderm (VE) expressing Apob and Amn [38], epiblast (EPI) expressing Pou5f1 and Nanog [39], extraembryonic ectoderm or ectoplacental cone (EXE/EPC) expressing Elf5 and Cdx2 [39], and trophoblast giant cells (TGC) expressing Gata2 [37] and Prl3d1 [40] (Fig. 4B–E).

By using the CellChat software, we predicted the ligand-receptor interactions between PDZ (S3) and TGC. We found a total of 20 ligand-receptor interaction pairs (Fig. 4F). Based on pathway analysis, these ligand-receptor interactions were enriched among ECM-receptor interaction (FDR = 1.00 × 10^–18^), PI3K-Akt signaling pathway (FDR = 1.00 × 10^–18^), Regulation of actin cytoskeleton (FDR = 1.26 × 10^–9^), Rap1 signaling pathway (FDR = 1.58 × 10^–6^), Ras signaling pathway (FDR = 7.94 × 10^–5^), MAPK signaling pathway (FDR = 1.26 × 10^–4^), Phospholipase D signaling pathway (FDR = 7.94 × 10^–3^) and Cytokine-cytokine receptor interaction (FDR = 3.98 × 10^–2^) (Fig. 4G).

### The impact of PDZ stromal cells on other stromal cells

**Fig. 5:**
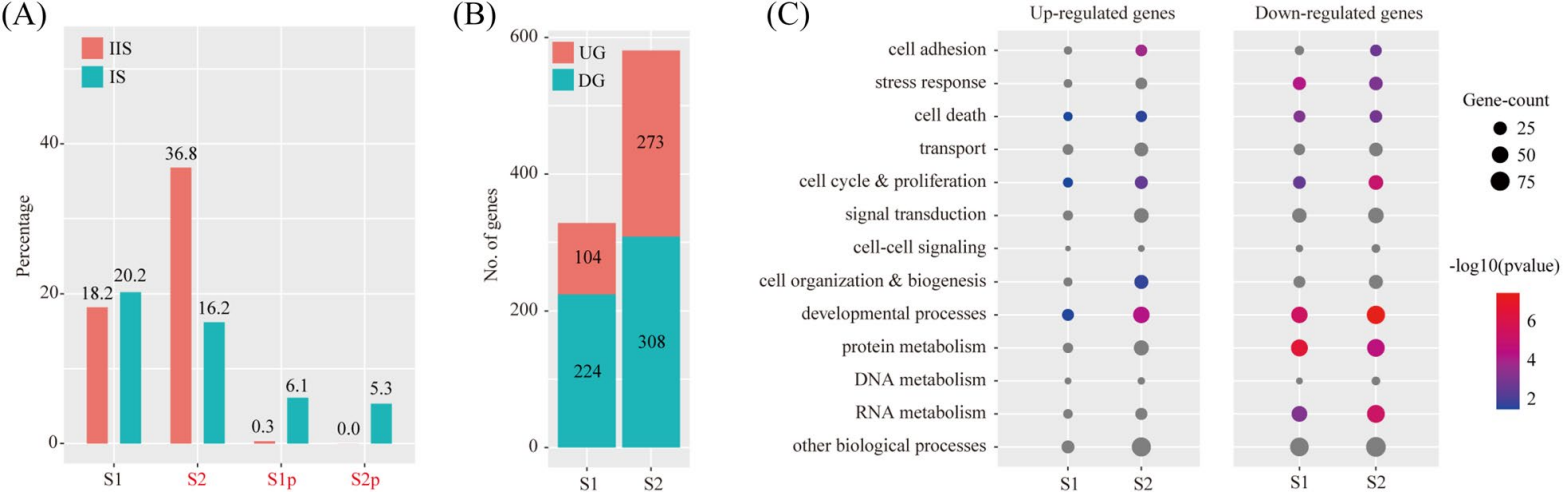
Cell abundance and gene expression changes in stromal cells upon embryo implantation. **A** Bar plot showing the cell population change of major stromal cell types (S1 and S2) at IS compared to IIS. Cell types with FC > 2 and P < 0.05 by χ^2^ test were labeled in red. **B** Bar plot showing the count of differentially expressed genes in each stromal cell type. The threshold values for differentially expressed genes were: logFC > 0.25 and P < 0.05. UG, up-regulated gene; DG, down-regulated genes. **C** Gene ontology (GO) enrichment analysis of differentially expressed genes. Differentially expressed genes were grouped based on MGI GOslim terms under the biological process categories. Up-regulated genes and down-regulated genes were tested separately. Abbreviations for cell types are listed in Fig. 2

We investigated the abundance of major stromal cell types at IS compared to IIS. The χ^2^ test was employed to assess the significance of difference between two groups. By using the criteria of P < 0.05 and fold change > 2, we found that the proportion of S1 was unchanged, whereas the proportion of S2 significantly decreased in IS compared to IIS. Meanwhile, S1p and S2p were almost exclusively detected in IS (Fig. 5A), and so was S3 (0.0% in IIS vs 3.1% in IS, data not shown). According to our trajectory analysis, S2p was originated from S2 and S3 was originated from S2p. Surprisingly, the proportion of all these S2-lineage cells (S2 + S2p + S3) together in IS was still less than that of S2 in IIS (24.6% vs 36.8%). This phenomenon was likely due to cell loss at PDZ from GD6. In fact, PDZ disappears by GD8 [41].

We investigated the breadth of transcriptional changes in each stromal cell type by performing differential gene expression analysis. Using a logFC cutoff of 0.25 and a pvalue cutoff of 0.05, we identified 328 and 581 differentially expressed genes for S1 and S2, respectively (Fig. 5B and Additional file 3: Table S3). We then explored the biological implications of differentially expressed genes using gene ontology (GO) analysis. Our results indicated that similar functional changes occurred during embryo implantation in both S1 and S2 (Fig. 5C).

**Fig. 6:**
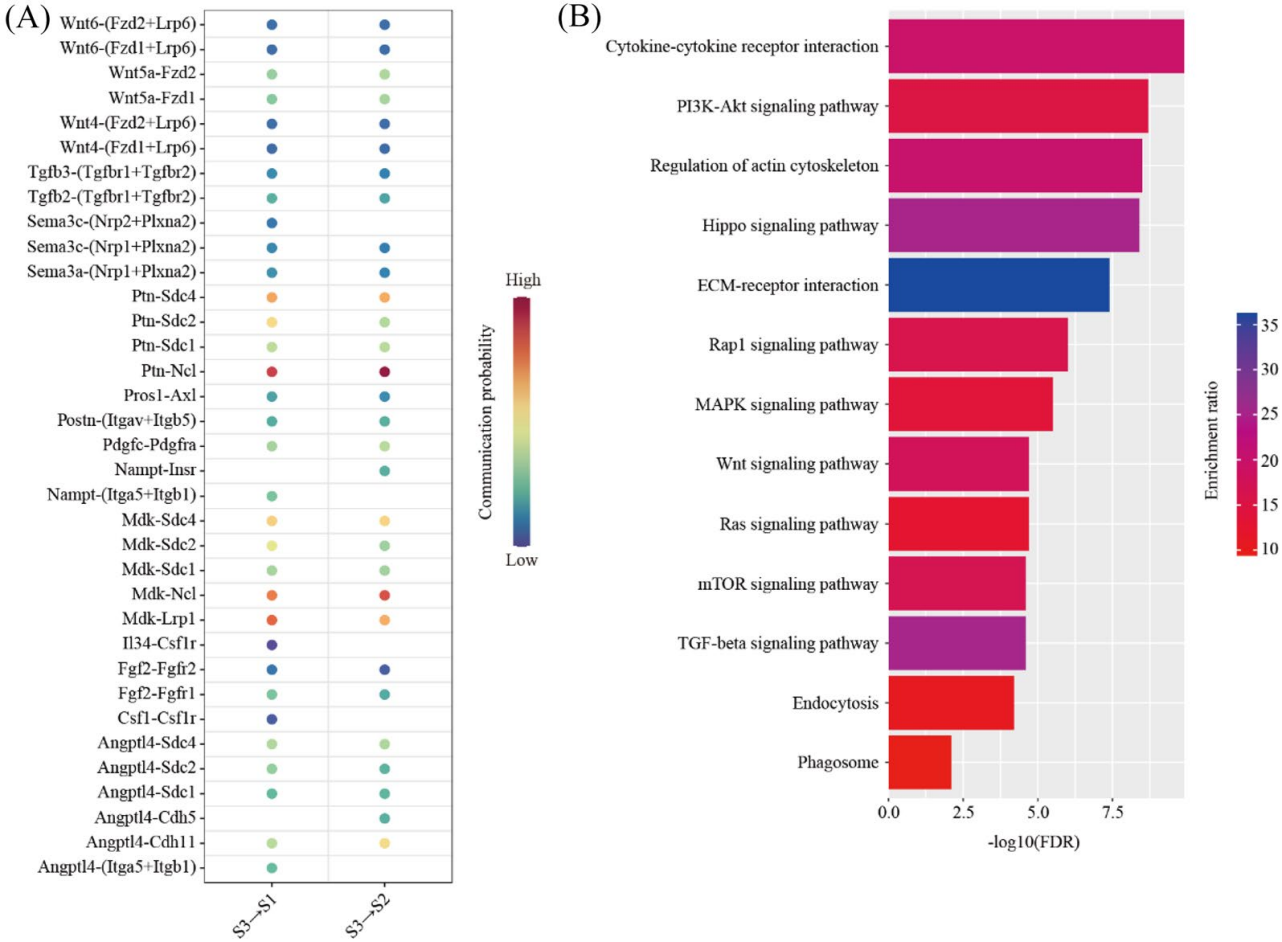
The impact of primary decidual zone stromal cells on other stromal cells. **A** Dot plot showing selected ligand-receptor interactions underlying the crosstalk between primary decidual zone stromal cells (S3) and other stromal cells (S1 and S2). The communication probability defined by the CellChat software were indicated by color. **B** KEGG Pathway enrichment analysis of ligand-receptor pairs by using the Metascape online tools

In order to determine the impact of PDZ (S3) on S1 and S2, we used the CellChat software to predict the ligand-receptor interactions. Only secreted factors from PDZ were considered. We found a total of 35 ligand-receptor interaction pairs (Fig. 6A). Pathway analysis revealed that these ligand-receptor interactions were enriched among Cytokine-cytokine receptor interaction (FDR = 1.26 × 10^–10^), PI3K-Akt signaling pathway (FDR = 2.00 × 10^–9^), Regulation of actin cytoskeleton (FDR = 3.16 × 10^–9^), Hippo signaling pathway (FDR = 3.98 × 10^–9^), ECM-receptor interaction (FDR = 3.98 × 10^–8^), Rap1 signaling pathway (FDR = 1.00 × 10^–6^), MAPK (FDR = 3.16 × 10^–6^), Wnt signaling pathway (FDR = 2.00 × 10^–5^), Ras signaling pathway (FDR = 2.00 × 10^–5^), mTOR signaling pathway (FDR = 2.51 × 10^–5^), TGF-beta signaling pathway (FDR = 2.51 × 10^–5^), Endocytosis (FDR = 6.31 × 10^–5^) and Phagosome (FDR = 7.94 × 10^–3^) (Fig. 6B).

### The impact of primary decidual zone stromal cells on immune cells

**Fig. 7:**
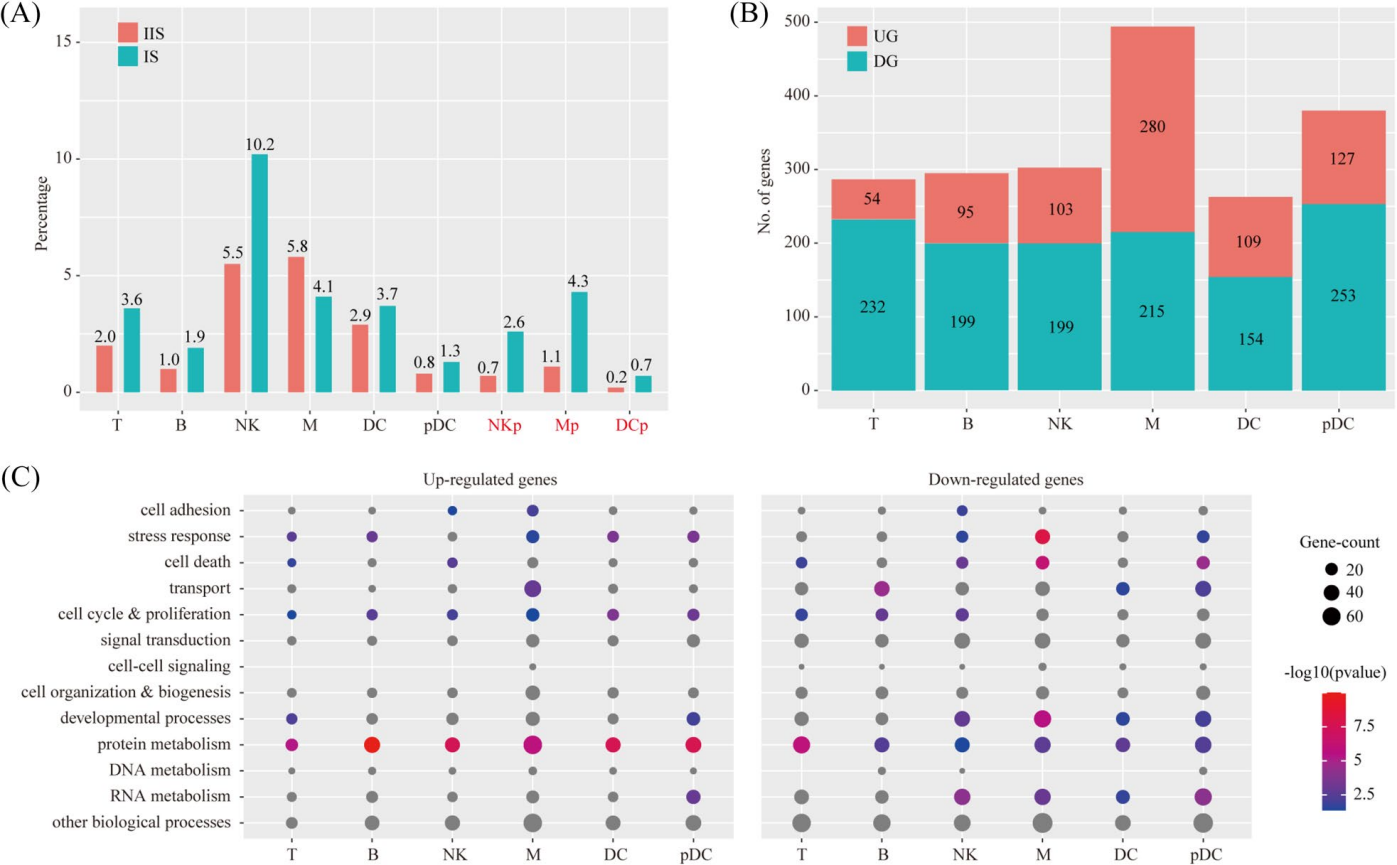
Cell abundance and gene expression changes in immune cells upon embryo implantation. **A** Bar plot showing the cell population change of major immune cell types (T, NK, B, M, DC and pDC) at IS compared to IIS. Cell types with FC > 2 and P < 0.05 by χ^2^ test were labeled in red. **B** Bar plot showing the count of differentially expressed genes in each immune cell type. The threshold values for differentially expressed genes were: logFC > 0.25 and P < 0.05. UG, up-regulated gene; DG, down-regulated genes. **C** Gene ontology (GO) enrichment analysis of differentially expressed genes. Differentially expressed genes were grouped based on MGI GOslim terms under the biological process categories. Up-regulated genes and down-regulated genes were tested separately. Abbreviations for cell types are listed in Fig. 2

By using the criteria of P < 0.05 and fold change > 2, we found that the proportion of NKp, Mp and DCp were significantly increased at IS compared IIS (Fig. 7A). Using a logFC cutoff of 0.25 and a pvalue cutoff of 0.05, we identified 286, 294, 302, 495, 263 and 380 differentially expressed genes for T, B, NK, M, DC and pDC respectively (Fig. 7B and Additional file 3: Table S3). Differentially expressed genes were further characterized by GO analysis (Fig. 7C). Our data indicated that each immune cell type invoked distinct biological processes in order to accommodate embryo implantation.

**Fig. 8:**
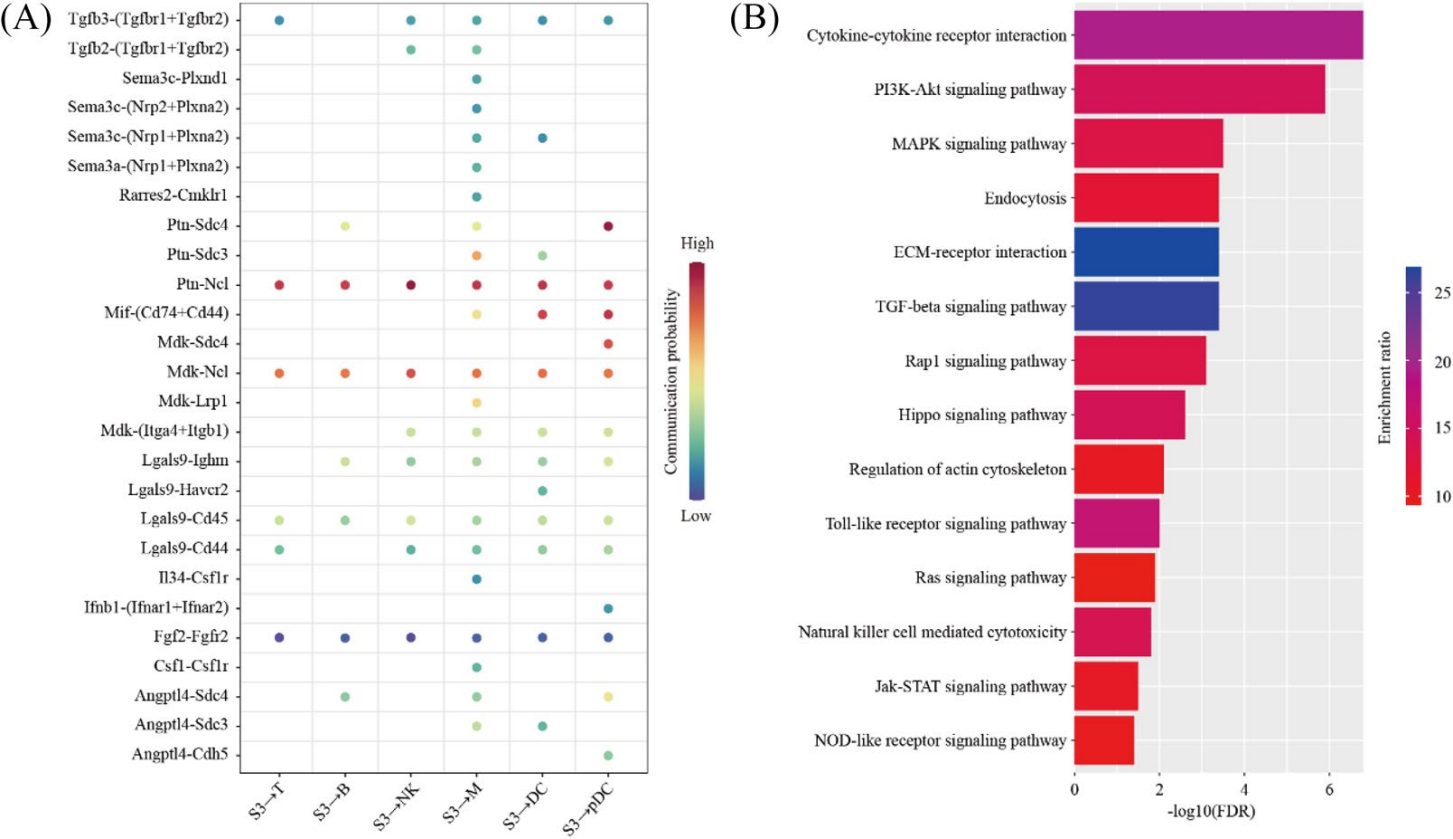
The impact of primary decidualization zone cells on immune cells. **A** Dot plot showing selected ligand-receptor interactions underlying crosstalk between primary decidualization zone cells (S3) and immune cells (T, NK, B, M, DC and pDC). The communication probability defined by the CellChat software were indicated by color. **B** KEGG Pathway enrichment analysis of ligand-receptor pairs by using the Metascape online tools

We predicted the ligand-receptor interactions between PDZ (S3) and immune cells (T, B, NK, M, DC and pDC) using the CellChat software. We found a total of 26 ligand-receptor interaction pairs (Fig. 8A). Pathway analysis revealed that these ligand-receptor interactions were enriched among Cytokine-cytokine receptor interaction (FDR = 1.58 × 10^–7^), PI3K-Akt signaling pathway (FDR = 1.26 × 10^–6^), MAPK signaling pathway (FDR = 3.16 × 10^–4^), Endocytosis (FDR = 3.98 × 10^–4^), ECM-receptor interaction (FDR = 3.98 × 10^–4^), TGF-beta signaling pathway (FDR = 3.98 × 10^–4^), Rap1 signaling pathway (FDR = 7.94 × 10^–4^), Hippo signaling pathway (FDR = 2.51 × 10^–3^), Regulation of actin cytoskeleton (FDR = 7.94 × 10^–3^), Toll-like receptor signaling pathway (FDR = 1.00 × 10^–2^), Ras signaling pathway (FDR = 1.26 × 10^–2^), Natural killer cell mediated cytotoxicity (FDR = 1.58 × 10^–2^), Jak-STAT signaling pathway (FDR = 3.16 × 10^–2^) and NOD-like receptor signaling pathway (FDR = 3.98 × 10^–2^) (Fig. 8B).

### The impact of primary decidual zone on endothelial cells

**Fig. 9:**
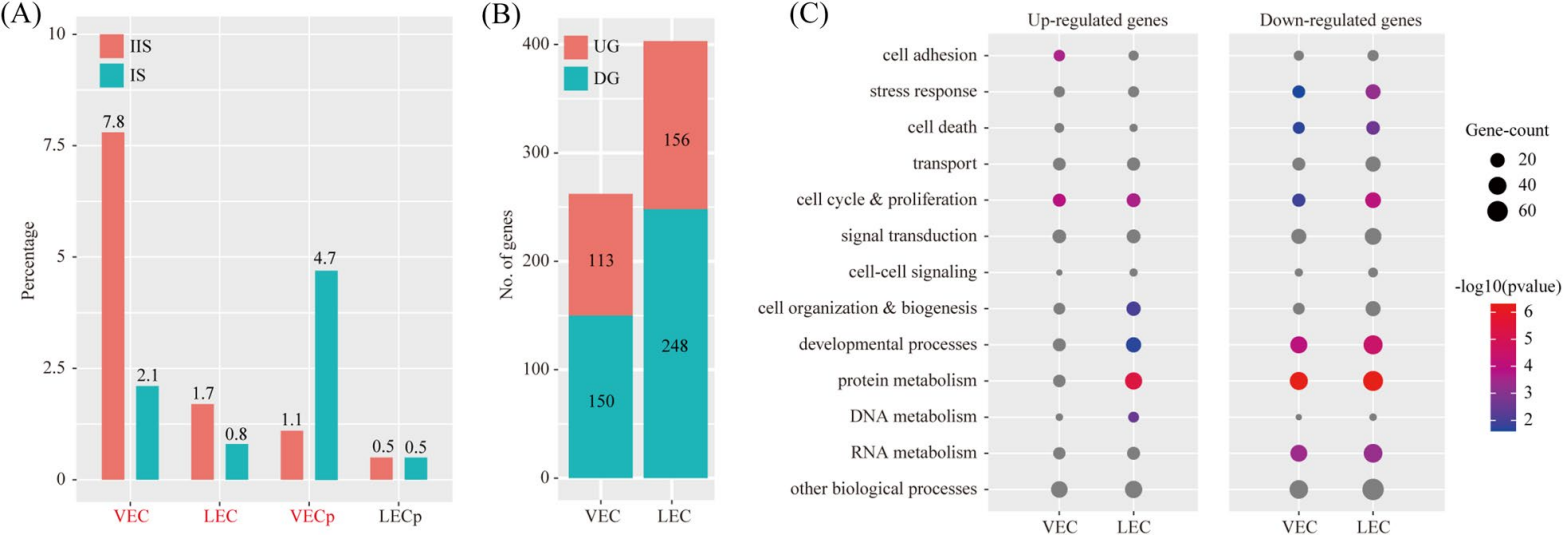
Cell abundance and gene expression changes in endothelial cells upon embryo implantation. **A** Bar plot showing the cell population change of major endothelial cell types (VEC and LEC) at IS compared to IIS. Cell types with FC > 2 and P < 0.05 by χ^2^ test were labeled in red. **B** Bar plot showing the count of differentially expressed genes in each immune cell type. The threshold values for differentially expressed genes were: logFC > 0.25 and P < 0.05. UG, up-regulated gene; DG, down-regulated genes. **C** Gene ontology (GO) enrichment analysis of differentially expressed genes. Differentially expressed genes were grouped based on MGI GOslim terms under the biological process categories. Up-regulated genes and down-regulated genes were tested separately

By using the criteria of P < 0.05 and fold change > 2, we found that the proportions of both VEC and LEC were significantly decreased in IS compared to IIS, which is in line with the fact that PDZ is avascular [42–44]. Interestingly, the proportion of VECp was significantly increased in IS compared to IIS, indicating that the PDZ is inducer of angiogenesis in the uterus (Fig. 9A). Using a logFC cutoff of 0.25 and a P value cutoff of 0.05, we identified 263 and 404 differentially expressed genes for VEC and LEC, respectively (Fig. 9B and Additional file 3: Table S3). These differentially expressed genes were further characterized by GO analysis (Fig. 9C).

**Fig. 10:**
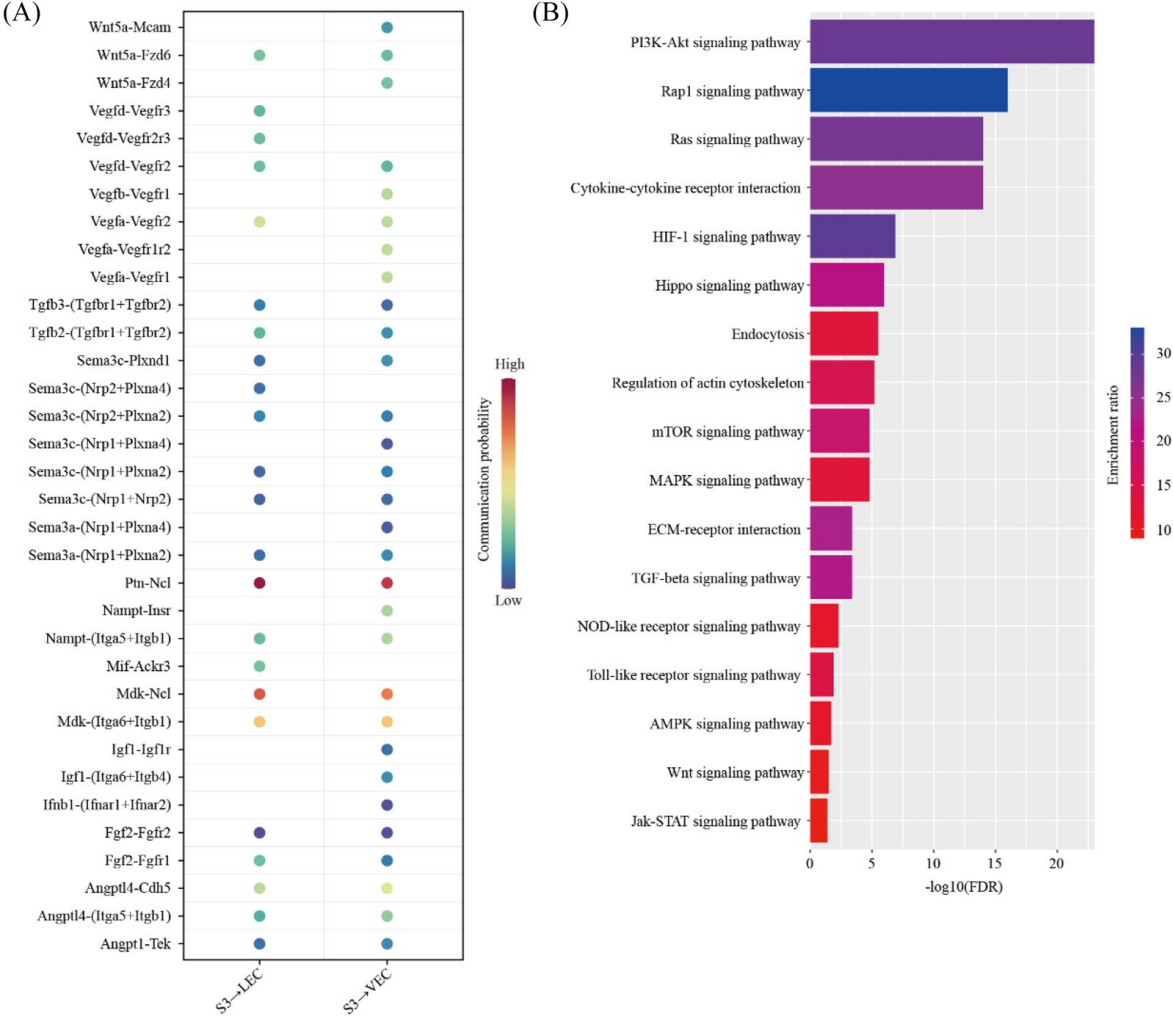
The impact of primary decidual zone on endothelial cells. **A** Dot plot showing selected ligand-receptor interactions underlying the crosstalk between primary decidualization zone cells (S3) and endothelial cells (VEC and LEC). The communication probability defined by the CellChat software were indicated by color. **B** KEGG Pathway enrichment analysis of ligand-receptor pairs by using the Metascape online tools

We predicted the ligand-receptor interactions between PDZ (S3) and VEC/LEC. We found a total of 34 ligand-receptor interaction pairs (Fig. 10A). Pathway analysis revealed that these ligand-receptor interactions were enriched among PI3K-Akt signaling pathway (FDR = 1.00 × 10^–23^), Rap1 signaling pathway (FDR = 1.00 × 10^–16^), Ras signaling pathway (FDR = 1.00 × 10^–14^), Cytokine-cytokine receptor interaction (FDR = 1.00 × 10^–14^), HIF-1 signaling pathway (FDR = 1.26 × 10^–7^), Hippo signaling pathway (FDR = 1.00 × 10^–6^), Endocytosis (FDR = 3.16 × 10^–6^), Regulation of actin cytoskeleton (FDR = 6.31 × 10^–6^), mTOR signaling pathway (FDR = 1.58 × 10^–5^), MAPK signaling pathway (FDR = 1.58 × 10^–5^), ECM-receptor interaction (FDR = 3.98 × 10^–4^), TGF-beta signaling pathway (FDR = 3.98 × 10^–4^), NOD-like receptor signaling pathway (FDR = 5.01 × 10^–3^), Toll-like receptor signaling pathway (FDR = 1.26 × 10^–2^), AMPK signaling pathway (FDR = 2.00 × 10^–2^), Wnt signaling pathway (FDR = 3.16 × 10^–2^) and Jak-STAT signaling pathway (FDR = 3.98 × 10^–2^) (Fig. 10B).

## Discussion

Embryo implantation is a crucial step for human embryo implantation. Previously, we performed single-cell RNA analysis of the mouse uterus during the apposition phase and attachment phase of embryo implantation on gestational days (GD) 4–5 [16, 17]. Here, by using the mouse as an animal model, we profiled the single-cell transcriptome for 16,257 cells from mouse uterus at the invasion phase on GD6. We revealed 23 distinct cell clusters, including 5 stromal cell clusters, 2 epithelial cell clusters, 1 smooth muscle cell cluster, 2 pericyte clusters, 4 endothelial cell clusters, and 9 immune cell clusters. To the best of our knowledge, the present study is the first to highlight the transcriptome landscape associated with the invasion phase of embryo implantation at single-cell resolution.

In order to accommodate embryo implantation, an important change in mouse uterus is the formation of primary decidual zone (PDZ). The first sign of PDZ formation occurs on the afternoon of GD5. PDZ is fully established by GD6 [45–47]. In this study, we found that the stromal cells at IIS can be divided into 2 cell types, superficial stromal cells expressing Hand2 and deep stromal cells which are negative for Hand2. Through pseudotime trajectory analysis, we confirmed that PDZ at IS was derived from superficial stromal cells. Superficial stromal cells start proliferate to become intermediate PDZ cells with high expression of Wnt4, Bmp2 and Mki67. Notably, Prl8a2, the marker for secondary decidual zone [36], was not expressed. These cells ceased proliferation and became PDZ cells [41]. The expression of another decidual marker Bmp2 [48] was reduced during this process. Interestingly, although PDZ cells expressed mesenchymal marker Vim, they were also positive for epithelial marker Krt19. Our result is in line with previous findings showing that PDZ is avascular and epithelioid in nature [42–44]. In fact, PDZ is thought to function as a partial permeability barrier to safeguard the implanting embryo, as only molecules smaller than 45 kDa are freely permeable though PDZ [49].

The trophectoderm (TE) of mouse embryo is created by the end of the pre-implantation period. After implantation, TE develops into two different types, polar TE and mural TE. Polar TE differentiates into extraembryonic ectoderm (ExE) and ectoplacental cone (EPC), while mural TE differentiates into trophoblast giant cells (TGC). TGC cells are in closest contact to the uterus during embryo implantation [50]. Thus, the interaction between TGC and PDZ represents the key mechanism of embryo implantation. Notably, PDZ cells expressed Ptn and Mdk, while the corresponding receptor Ncl was expressed in TGC. Currently, little is known about the role of Ptn and Mdk in regulating embryo implantation. Additionally, we found that Fgf2 and Angptl4 expressed in PDZ might function via their receptors Fgfr2 and Cdh5 expressed in TGC. In mouse uterus, Fgf2 in the superficial stromal cells maintains proliferation of epithelial cells [32]. Because Fgfr2 is also expressed in TGC, Fgf2 in PDZ might contribute to the proliferation of TGC. Angptl4 acts as a secretory protein to regulate angiogenesis in various tissues including the uterus [51]. Vascular endothelial cadherin Cdh5 is also expressed trophoblast cells [52]. Our findings supported the findings showing that predecidual stromal cells have distinctive characteristics of pericytes [53] and trophoblasts mimic the endothelial cells [54]. On the other hand, TGC cells expressed Pdgfa, while the corresponding receptors Pdgfra and Pdgfrb were expressed in PDZ. Indeed, proteome profiling confirmed the presence of Pdgfa in trophoblast supernatant [55]. Pdgfra and Pdgfrb are regarded as canonical cell markers for uterine stromal cells [30]. Therefore, TGC might regulate proliferation and differentiation of PDZ via Pdgfa. Apart from secreted signaling, we found that TGC might also crosstalk with PDZ through ECM-receptor interaction. Our data provided clues for the mechanisms underlying embryo implantation from the aspect of cell–cell communication.

Besides the emergence of PDZ, by comparing IS with IIS, we found apparent cell type abundance changes upon embryo implantation. In particular, the proliferating subsets of S1, S2, NK, M, DC and VEC were significantly increased. There were also massive gene expression changes in these cell types. Considering spatial relationships between cell types, uterine cell types other than PDZ are unable to directly communicate with embryonic cells during embryo implantation. Therefore, these changes were likely caused by PDZ indirectly via secreted signaling. Indeed, we found that many soluble factors from PDZ, such as Wnt4, Wnt5a and Wnt6, might have an influence on S1 and S2 cells. Wnt4 is most abundant in the decidual cells. In uterus-specific Wnt4 knockout mice, the embryos were able to attach to the uterine luminal epithelium, but they failed to successfully invade into the uterine stroma [33]. During embryo implantation, Wnt5a is highly localized in stromal cells at the mesometrial pole. Mice with uterine inactivation of Wnt5a show impaired embryo implantation and decidualization [47]. Using Wnt6-null mice, it has been shown that Wnt6 is critical for normal stromal cell proliferation during decidualization [56]. The idea that Wnt signaling might act in a paracrine way between PDZ and other stromal cells deserves further investigation. Additionally, PDZ-secreted Tgfb2 and Tgfb3 might target immune cells. Immune cells accumulate at embryo implantation sites and some of them such as DC cells play an important role in embryo implantation [57]. Increasing evidence supports the involvement of TGFβ signaling in uterine function including embryo implantation [58]. However, whether the TGFβ-mediated PDZ-DC interaction is functional during embryo implantation is still unknown. Moreover, we found that PDZ-secreted Vegfa, Vegfb and Vegfd might target endothelial cells. PDZ is avascular, but angiogenesis is accompanied with the emergence of SDZ. It has been well established that uterine immune cells are responsible to angiogenesis in SDZ [59]. Thus, we suggested that PDZ, by synthesizing VEGFs, might be another inducer of angiogenesis besides immune cells. Altogether, our data highlighted the role of PDZ as a key mediator for uterine response in different cell types during embryo implantation.

In conclusion, this study provided a comprehensive single-cell transcriptome atlas for mouse uterus at the invasion phase of embryo implantation. Our data present a valuable resource for deciphering the molecular mechanism underlying embryo implantation.

## Supplementary Information

**Additional file 1: Table S1.** The complete list of novel and known marker genes for each cell type.

**Additional file 2: Table S2.** The complete list of signature genes for stromal cell clusters.

**Additional file 3: Table S3.** The complete list of differentially expressed genes in IS compared to IIS for all cell types (logFC > 0.25 and P < 0.05).

## Data Availability

The datasets used and/or analysed during the current study are available from the corresponding author on reasonable request.
